# The impact of β-glucan yeast extract treatment on melanoma development, tumor-cell deposit infiltration, and immune response

**DOI:** 10.3389/fimmu.2026.1752221

**Published:** 2026-04-20

**Authors:** Bruno Miranda dos Santos Oliveira, Fernanda Paloma Duarte Trierweiler, Bianca Ramos Mesquita, Jose Nathan Andrade Muller Da Silva, Washington Luís dos Santos, José Mengel, Fabíola Cardillo

**Affiliations:** 1 Laboratory of Structural and Molecular Pathology (LAPEM), Gonçalo Moniz Institute, Oswaldo Cruz Foundationl, Salvador, Bahia, Brazil; 2 Immunology Program, Federal University of Bahia (UFBA), Salvador, Bahia, Brazil; 3 Oswaldo Cruz Institute, Oswaldo Cruz Foundation (Fiocruz), Rio de Janeiro, Brazil; 4 Petrópolis Medical School, FMP-FASE, Petrópolis, Rio de Janeiro, Brazil

**Keywords:** adjuvant therapies, experimental melanoma, immunomodulation, trained immunity, β-glucan

## Abstract

**Introduction:**

Melanoma is one of the most aggressive types of tumors, and strategies for modulating the immune response have been explored as adjuvant therapies. One notable candidate is β-glucan, a polysaccharide derived from Saccharomyces cerevisiae *(S. cerevisiae)*, known for its immunomodulatory properties. However, its effects on experimental melanoma have not yet been fully understood.

**Objective:**

This study aims to evaluate the treatment effects of β-glucan contained in an extract from *S. c.* (β-GESc) in the B16F10 melanoma model to assess its impact on tumor development.

**Methods:**

C57BL/6 mice were treated with β-GESc, and evaluations were conducted at different time points during tumor progression (days post-inoculation, or d.p.i). Flow cytometry was used to characterize splenic cell populations and cytokines, and histopathological assessments were performed to evaluate spleen structure. Hematological analysis was performed to assess the peripheral blood.

**Results:**

β-GESc-treatment increased spleen size and the absolute number of splenocytes, including macrophages, dendritic cells (DCs), NK cells, and NKT cells. Additionally, it enhanced MHC class II expression by DCs and promoted the formation of germinal centers, indicating immune activation in the spleen. The treatment also increased monocyte and lymphocyte counts, improved survival rates, and reduced tumor growth. Treated animals preserved the white pulp region of the spleen and showed an expansion of the T-cell zone (PALS) at 18 d.p.i., whereas untreated mice exhibited tumor cell infiltration in the spleen at 24 d.p.i. Furthermore, treated animals displayed higher absolute numbers of CD4+ and CD8+ T cells producing IFN-γ and TNF-α, particularly after anti-CD3 stimulation.

**Conclusions:**

Treatment with β-GESc demonstrates immunomodulatory potential by increasing both splenic and systemic cell frequencies, contributing to the control of experimental melanoma.

## 1 Introduction

Melanoma is a skin tumor originating from genetic and molecular alterations in melanocytes, cells derived from the neural crest during embryonic development (1). These cells primarily reside in the basal layer of the epidermis and perform the essential function of synthesizing melanin in melanosomes, contributing to skin pigmentation (2). Among the factors associated with melanoma development, exposure to ultraviolet (UV) light stands out, with a complex relationship to the disease (3). UV radiation can cause significant DNA damage, leading to multiple somatic mutations that affect fundamental biological processes, such as cell proliferation, the cell cycle, and apoptosis (4). This damage initiates a cascade of molecular events, including the elevation of reactive oxygen species (ROS) due to oxidative stress, which activates inflammatory signaling pathways essential for tumor progression (5).

Accurate diagnosis and melanoma classification are key to determining prognosis and treatment strategy (6). Although histopathology remains the gold standard, complementary methods, such as analysis of clinical and epidemiological characteristics, immunohistochemistry (IHC) for identification of melanocyte-specific markers or antigens, and genomic tumor profiling, provide valuable additional insights (7). Skin biopsy, widely used to diagnose cutaneous melanoma, confirms the diagnosis and assesses the depth of invasion using the “Breslow” criteria (8). This measure is a crucial prognostic indicator for determining the optimal therapeutic approach, as it correlates with the thickness of the primary lesion in human beings (9).

The overall survival rate is favorable in the early stages of the disease, with 5-year survival rates of 94% to 98% for melanoma *in situ* or stage I. However, survival drops drastically in more advanced stages (III-IV), falling from 10% to 15% (10). Surgical treatment is highly effective in localized disease (stages I and II), involving the excision of the primary lesion and, if indicated, removal of the sentinel lymph node in cases of lymph node metastasis (11). However, it is known that this approach has limitations for advanced melanoma due to the high mutation rate and the tumor’s ability to evade the immune response. This challenge has led to the exploration of novel therapeutic strategies for melanoma (12). In this scenario, β-GE*Sc* emerges as a strategic ally to combat the innate or acquired resistance observed in patients treated with checkpoint inhibitors, such as anti-PD-1 and anti-CTLA-4, which frequently fail to induce lasting responses in cases of high refractoriness (13, 14). This immunomodulatory profile resulted in greater control of tumor growth and a significant improvement in overall survival.

Recent immunological studies have highlighted the heterologous effects of vaccines, demonstrating potent, nonspecific immune responses that extend beyond the initial target and result in significant, nonspecific crossover responses against the tumor microenvironment (TME) (15). These studies support the development of trained immunity mechanisms to confront the tumor microenvironment (TME), inducing potent effector responses against tumors, primarily mediated by myeloid cells. Trained immunity, mediated by myeloid cells, enhances effector responses against tumors and plays a pivotal role in inducing adaptive antitumor immunity. This innate mechanism plays a crucial role in inducing adaptive cellular immunity against tumors (16).

Notably, recent studies have challenged the notion that only the adaptive system generates long-term memory, revealing that certain substances can induce lasting changes in the innate immune response, thereby enhancing its capacity and effector potency mediated by trained immunity (17). This mechanism is characterized by an increase in the nonspecific response, involving epigenetic reprogramming, transcriptomic rewiring, and metabolic. Some vaccines used as primary changes that enhance the immunological response, especially in the face of secondary challenges (18). A central mechanism in this process includes histone modifications and chromatin reconfiguration, alongside DNA methylation, expression of long non-coding RNAs (lncRNAs), and modulation of microRNA, and modulation in the persistence, all of which can alter intracellular signaling in innate cells, inducing changes in cellular metabolism (17). Some vaccines used as primary stimuli were associated with these typical mechanisms of trained immunity (19). These studies observe changes in the dynamics of the immune response in several cells, including monocytes, macrophages, dendritic cells (DCs), neutrophils, NK cells, and innate lymphoid cells (ILCs), through a process that results in an increase in effector capacity, including the production of pro-inflammatory cytokines, as well as an increase in ROS and antimicrobial properties (20–22). Such modifications, mainly observed *in vitro*, have been demonstrated using vaccines such as live attenuated tuberculosis (MTBVAC) (23), oral measles vaccine (24), smallpox vaccine (25), β-glucans (21), and bacille Calmette-Guérin (BCG) (20). BCG is even used as a nonspecific immunotherapy in bladder cancer and demonstrates anti-tumor effects that help mitigate tumor progression through processes such as direct action on cancer cells, causing cytokine release or induction of apoptosis through caspase-dependent pathways or through mechanisms associated with trained immunity (17, 26, 27). The effects of these applications are notable, mainly in monocytes and macrophages (18). With this, cells of the innate immune system can adapt to respond to infections and tumors independently of specific antigens (15).

Yeast-derived products, particularly β-glucans, have attracted increasing attention for their antioxidant and immunological properties (28). β-1,3-glucan, present in cell wall extracts, is a component whose degree of chain polymerization is around 1,500 glucose units long, with β-1,6 bonds between chains that guarantee branching and may also be covalently linked to chitin (29). Although β-glucans are known to influence host immune responses, significant gaps remain in understanding how the structural aspects of the yeast cell wall relate to its immunomodulatory effects, specifically the mechanisms and effectors involved in the immune response (30). In fact, β-glucans exhibit multiple immunomodulatory functions, including antioxidant properties and enhanced cellular and antineoplastic immunity, which may target tumor cells (31).

In this work, we evaluated cells with anti-tumor potential in an experimental melanoma model and assessed structural changes before and after administration of Saccharomyces cerevisi*ae* cell wall extract. Furthermore, we sought to determine whether yeast extract containing β-glucan (β-GESc) can modify the dynamics of the immune system’s anti-tumor response and increase animal survival, and whether splenectomy can impact tumor progression and anti-tumor responses.

## 2 Material and methods

### 2.1 Animals

4–6 week-old C57BL/6 mice derived from the Isogenic Animal Facility of the Gonçalo Moniz Institute, Fiocruz- Bahia, were used. All experimental procedures were approved by the Animal Use Ethics Committee (CEUA) of the Gonçalo Moniz Institute/Fiocruz (protocol CEUA 013-2023). Mice were divided into four main groups, as shown in **Supplementary Figure 1** (S1A).

### 2.2 Tumor cell inoculation

Tumor cells of the B16F10 lineage, widely used and described in the scientific literature, were kindly provided by Dr. Ricardo Brentani through the Ludwig Institute for Cancer Research (32) and maintained with serial passages in C57BL/6. The cells were thawed and placed in culture bottles containing RPMI medium (Gibco) supplemented with 10% inactivated fetal bovine serum (FBS, Gibco) at 37 °C and 5% CO2. Then, cell viability was assessed using 0.4% Trypan Blue (Gibco), and the cells were adjusted to 1x10^5^ cells and injected into the base of each animal’s ear, as described in Bonfim et al. (33). The B16F10 lineage can also be commercially acquired as described in (34). After inoculation, these cells were isolated *ex vivo* and rapidly cultured *in vitro*.

### 2.3 Tumor growth and mortality *in vivo*

The animals and tumor growth were monitored daily using a high-precision digital caliper. For euthanasia, animals that presented ulcerations at the tumor site or that showed growth greater than 01 centimeters (cm) were sacrificed. Mortality was also monitored daily. Initial sample sizes were set at n=10 for survival and tumor growth curves. For endpoint analyses at 18 and 24 days post-inoculation (d.p.i.), a subset of n=4 animals per group was randomly selected from survivors. This sample size was determined by the intrinsic mortality kinetics of the B16F10 model, ensuring statistical pairing and tissue viability in the untreated tumor group through the study’s final time point.

### 2.4 Administration of β-GE*Sc*

C57BL/6 mice weighing approximately 18-22g received a daily dose of 1 mg of yeast extract of *Saccharomyces cerevisiae* containing β-glucan (*Saccharomyces cerevisiae*, Florien, São Paulo, Brazil, CAS9041-22-9) from the point of tumor appearance (seventh day after inoculation of tumor cells). The β-1,3-glucan present in the extract used is a cell wall extract, with a degree of polymerization of around 1,500 glucose units/chain and β-1,6 linkages between chains that confer branching. It may also be covalently linked to chitin and contains >75% glucans and a smaller fraction of mannans, with antioxidant and immunological actions (28). The β-GE*Sc* dosage was set at 1 mg per animal (approximately 40–80 mg/kg), a concentration previously validated for its biological safety, lack of systemic toxicity, and ability to induce significant immune responses, as reviewed (35–38). The mice were monitored during and after the injection to observe possible adverse reactions. The control group received strictly the same volume (200μL) of pure PBS (without β-GE*Sc*), ensuring volumetric consistency across all groups.

### 2.5 Peripheral blood and selective cell counting

Blood from mice was collected through the submandibular vein to analyze hematological parameters using the PE7010vet analyzer (Henzhen Prokan Electronics Inc., Düsseldorf, Germany). The animals were manually immobilized, and the puncture was performed using a needle or lancet, applying firm pressure to the hairless point (located caudally to the eye and ventrally to the ear). Blood was collected in a 1.5 mL conical tube containing EDTA. Hemostasis was achieved by applying gauze pressure to the puncture site before returning the animal to the mini-isolator.

### 2.6 Cell culture, surface molecule labeling, and intracellular splenic cytokines

After counting, the splenic cells were added to 96-well U-shaped plates, with 2 × 10^6^ cells per well. The cells were then washed with FACS (flow cytometry) buffer, which consists of PBS, 5% inactivated FBS, and 0.1% sodium azide. Next, the cells were incubated with an Fc block (clone 2.4G2) for 15 minutes at 4°C in FACS buffer, as described by 39. The following monoclonal antibodies were added for labelling: anti-CD11b FITC (clone M1/70.15, Life Technologies, cat. RM2801, 1/50), anti-CD11c BV421 (HL3, BD Horizon, cat. 585452, 1/100), anti-F480 PE (clone BM8, BioLegend, cat. 123109, 1/100), anti-MHC-II APC (M5/114.15.2, Abcam, cat. ab93559, 1/400), anti-NK1.1 PE (BioLegend, cat 108707, 1/100), anti-CD3 PE (17A2, BioLegend, cat.100205, 1/100), anti-CD4 FITC (RM4-5, Invitrogen, cat. MDC0401, 1/100), anti-CD8 APC (5H10, Invitrogen, cat. MCD0805, 1/200). For intracellular cytokine detection, cells were adjusted to 2 × 10^6^ cells per well and placed in 24-well flat-bottom culture plates with 1 mL of cell suspension per well. The cells were then stimulated with anti-CD3 (5 µg/mL) and incubated in a humidified oven at 37°C with 5% CO2 for 24 hours. Following this incubation, Brefeldin A (Invitrogen) was added at 2.5 µg/mL, and the cells were incubated for an additional 6 hours. They were then transferred from the plate into 15 mL Falcon tubes containing complete RPMI medium. The tubes were centrifuged according to the specified settings. The antibodies of interest were added to each well containing 2 × 10^6^ cells/mL and incubated for 20–30 minutes at 4°C in a refrigerator. Then, after cell fixation and permeabilization, 100 µl of Cytofix solution (BD Biosciences) was added, washed twice with PermWash Buffer solution (100-200 µl) (BD Biosciences), incubated, and washed twice with FACS Buffer. Following that, anti-cytokine antibodies were used for intracellular labeling: IFN-γ-PE (XMG 1.2, Tonbo Biosciences, cat. 50-7311-U100) and TNF-α-PECY7 (MP6-XT22, BioLegend, cat. 506323), diluted in PermWash and then incubated at 4°C for 30 minutes. After this incubation, the cells were washed and transferred to a FACS tube containing 400 µl of 1x PBS. The samples were then analyzed on the LSRFortessa cytometer (BD Biosciences) to obtain readings for 200,000 events.

### 2.7 Immunohistochemistry and splenic conventional histology

Splenic tissue fragments were promptly retrieved from the midsection of the spleen by making a transverse incision across the capsule and the larger axis of the spleen. These fragments were then prepared for histological analysis, as described in Fontes et al. (40). Spleen fragments underwent fixation in formalin-acetic acid-alcohol solution, followed by embedding in paraffin. Tissue sections, 4 micrometers thick, were stained using hematoxylin and eosin. Furthermore, immunohistochemistry was employed for further characterization. The observations were validated through morphometric analysis, as described in Hermida et al. (41). Procedures: Deparaffinization and rehydration were performed in xylene, followed by a series of decreasing ethanol concentrations. Endogenous tissue peroxidase was blocked with 3% hydrogen peroxide, and antigen retrieval was performed using a Dako PT Link (PT100/PT101) high pH module at 97°C for 20 minutes. Diaminobenzidine was used as a chromogen. Sections were counterstained with hematoxylin. The primary antibodies, anti-CD3 polyclonal (produced in rabbit, Abcam, ab1669) or anti-CD20 (Abcam, ab62088), were added to the slides and incubated overnight (12–16 hours) at 4°C in a humid chamber. The following day, the slides were washed twice with 1x PBS for 5 minutes each. The secondary polymer conjugated with HRP (ImmPress, Vector Laboratories) was added, followed by incubation in an oven at 37°C for 30 minutes.

As a negative control, the sections were incubated simultaneously with immunoglobulins of the same isotype and species as the primary antibody. The analysis of the marked areas was conducted using ImageJ. To calculate the percentage, the following formula was used: (marked area/total area) × 100. The total number of cells in the area was determined by taking the average number of cells counted in subareas of the follicle, multiplying it by the total follicular area, and then multiplying by 1,000,000. Statistical analysis was used to compare the resulting values. All reactions were developed using a 3,3’-diaminobenzidine chromogen solution and counterstained with Harris hematoxylin. The density of leukocyte populations was estimated using morphometric analysis. For melanin detection, splenic fragments were fixed in PBS with a 10% (v/v) formaldehyde solution (Merck), embedded in paraffin, and the histological sections were stained with hematoxylin and eosin (H&E), Fontana-Masson (FM) for melanin detection, and Perls’ Prussian Blue (PPB) for analyzing iron deposition.

### 2.8 Statistical analysis and data evaluation

For data analysis, the normality of the sample distribution was assessed using the Shapiro-Wilk test in GraphPad Prism 8 software. The students’ t-test and ANOVA were used to test the differences between or among groups. The Mann-Whitney and Kruskal-Wallis tests were used for data that did not follow a normal distribution. To assess survival, the Log-Rank test was adopted. Furthermore, Kaluza software (Beckman Coulter) was used to analyze the flow cytometry results. P values ​​<0.05 were considered significant.

## 3 Results

### 3.1 The intraperitoneal treatment with β-glucan-enriched extract from *S. cerevisiae* improves animal survival and helps control melanoma local growth

**Figure 1:**
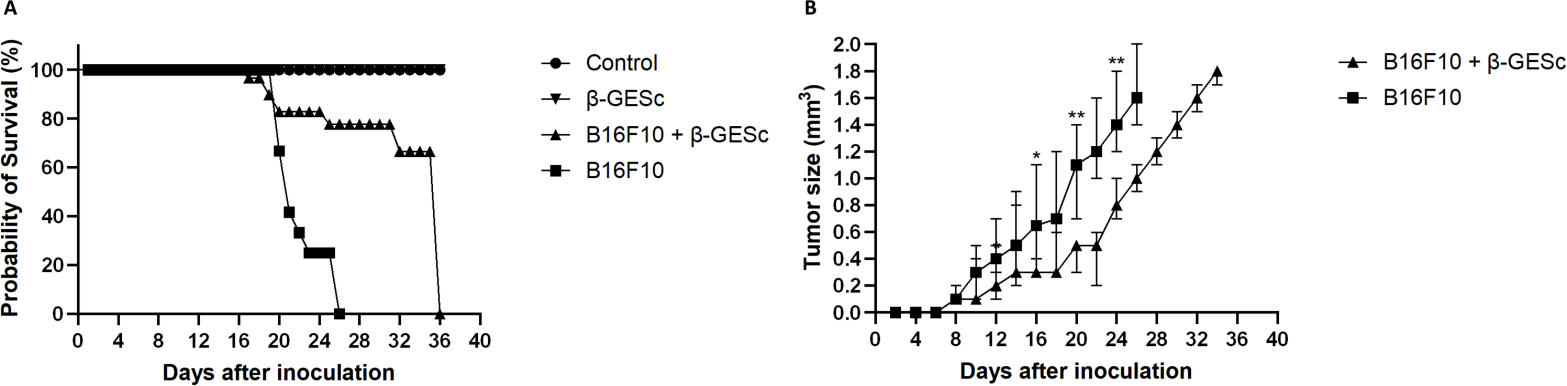
Analysis of survival and tumor growth in different experimental groups treated with β-GE*Sc*. **(A)** Survival curves analysis in the different experimental groups. The Log-rank test was used as the statistical method (p < 0.0001), with n = 10 per group. **(B)** Tumor size analysis in different experimental groups over time after B16F10 cell inoculation in C57BL/6 mice. The Kruskal-Wallis test was used to compare differences between groups at various time points, showing statistical significance at 12 days post-injection (d.p.i.). (p = 0.0011), 16 d.p.i. (p = 0.0117), 20 d.p.i. (p = 0.0037), and 24 d.p.i. (p = 0.0008). Dunn’s multiple-comparison tests revealed a significant difference between the β-GE*Sc*-treated and untreated tumor groups (p = 0.0034). Results are expressed as median values (SEM±) for each experimental group (n = 10 per group).

We first evaluated the effects of administering β-GE*Sc* in C57Bl/6 mice inoculated with melanoma cells in the ear’s pinna. Following the experimental protocol (**Supplementary File S1**), the sizes of the developing tumors and the splenic sizes of GESc-treated and untreated mice are shown in (**Supplementary Files S1B, S1C**). Our data indicate that β-GE*Sc* administration improved the clinical outcomes in the B16F10 melanoma model, as β-GESc-treated mice showed higher survival rates than the untreated control group (**Figure 1A**). The results also revealed that only animals treated with β-GE*Sc* developed significantly smaller tumors at various evaluation points (12, 16, 20, and 24 d.p.i), as shown in **Figure 1B**. An increased absolute number of splenocytes in animals treated with β-GE*Sc* is shown in **Table 1**, compared to untreated mice.

**Table 1:** Total splenic cell count in different experimental groups at 24 d.p.i.

Group	Total splenocytes (average)	SD±
Controle	67.000.000	16.552.945
β-GE*Sc*	150.250.000	53.501.558
B16F10 + β-GE*Sc*	145.750.000	11.420.012
B16F10	89.250.000	14.285.774

Total number of splenocytes counted in individual spleens within the experimental groups evaluated, using the Neubauer chamber. Results are presented as mean ± SD. N = 4/group, 24 d.p.i.

### 3.2 Splenic myeloid, lymphoid, and T cell-cytokine production in β-GE*Sc*-treated animals carrying B16F0

**Figure 2:**
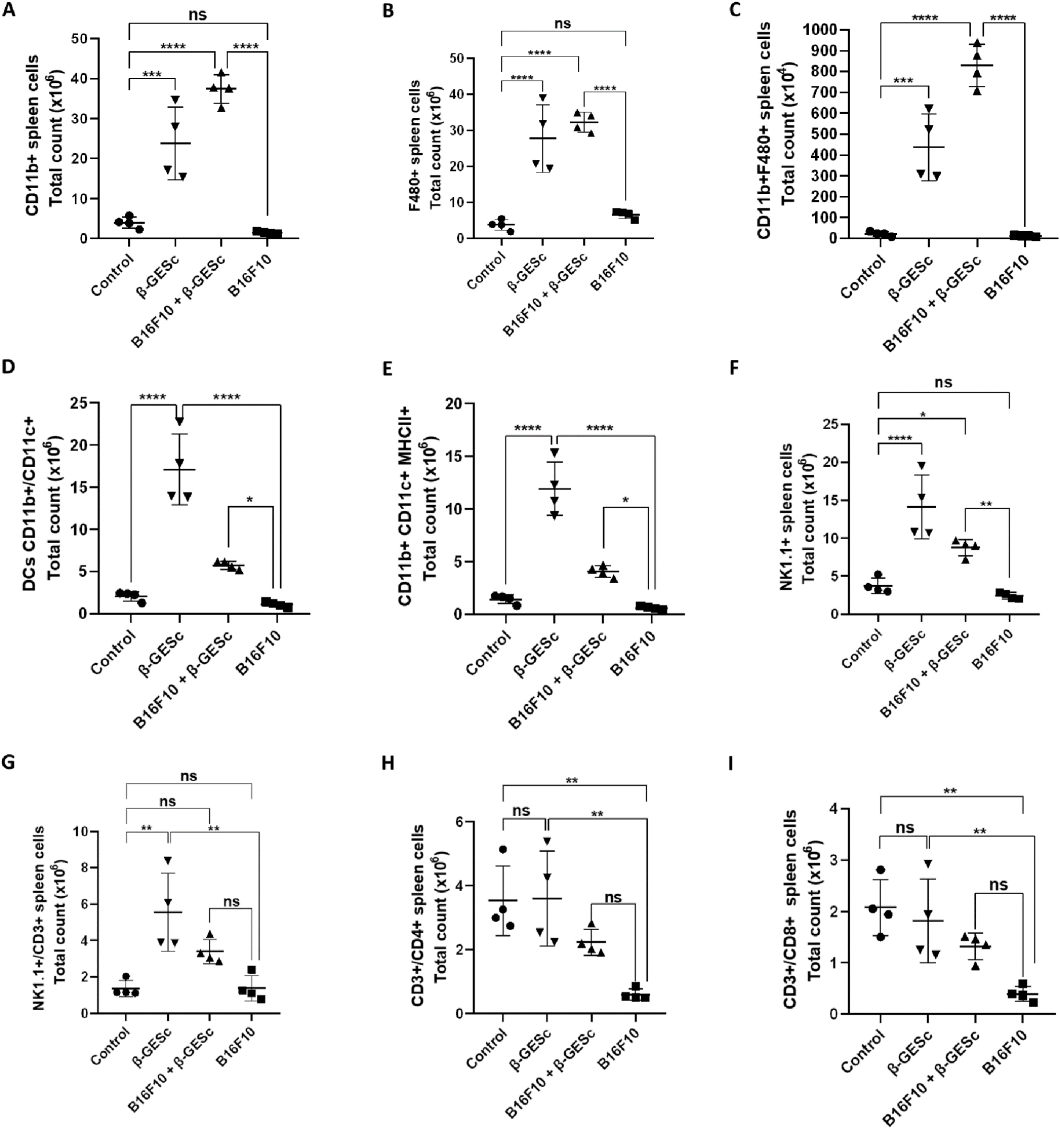
Analysis of the effect of β-GE*Sc* administration on absolute splenocyte counts at 24 days post-inoculation (d.p.i.). Absolute numbers of CD11b+ cells **(A)**, F4/80+ cells **(B)**, and double-positive CD11b+F4/80+ cells **(C)** are shown, with ANOVA p < 0.0001 and Tukey’s multiple comparison tests showing significance between B16F10+βGE*Sc* and B16F10 groups (p = 0.0001), indicated by (****). In **(D)**, the absolute number of dendritic cells (DCs; CD11b+CD11c+) is presented, with ANOVA p < 0.0001 and Tukey’s post-test p = 0.0419, indicated by (*). In **(E)**, the absolute number of DCs expressing MHC-II is shown, with ANOVA p < 0.0001 and Tukey’s post-test p = 0.0141, indicated by (*). In **(F)**, absolute NK1.1+ cell numbers are shown, ANOVA p < 0.0001 and Tukey’s post-test p = 0.0082, indicated by (**). In **(G)**, absolute numbers of NKT cells (CD3+NK1.1+) are presented, ANOVA p = 0.0009 and Tukey’s post-test p = 0.1347. In **(H)**, absolute CD3+CD4+ cell numbers are shown, ANOVA p = 0.0022 and Tukey’s post-test p = 0.1197. In **(I)**, absolute numbers of CD3+CD8+ cells are shown, ANOVA p = 0.0028 and Tukey’s post-test p = 0.1011. Statistical differences between groups were analyzed using a one-way ANOVA followed by Tukey’s multiple-comparison test. Results are expressed as mean ± SD. n = 4 per group.

**Figure 3:**
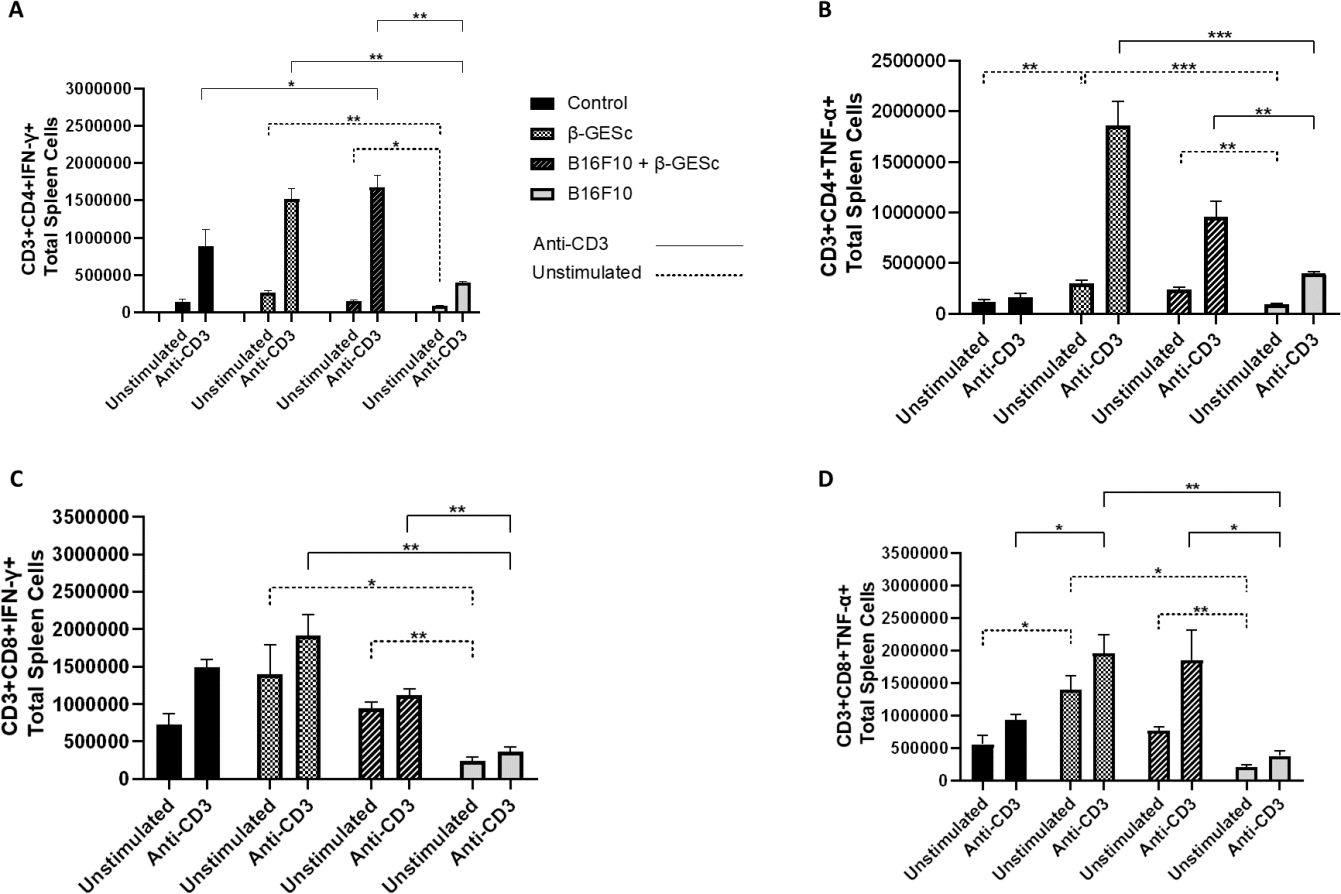
Evaluation of cytokine production by double-positives CD3+CD4+ and CD3+CD8+ splenocytes. In **(A)**, the absolute number of CD3+CD4+IFN-γ+ cells in spleen tissue. Sidak’s multiple comparison tests: B16F10+β**-**GE*Sc* vs. B16F10 = 0.0229 (unstimulated), indicated by (*), and = 0.0042 (anti-CD3-stimulated), indicated by (**). In **(B)**, the absolute number of CD3+CD4+TNF-α+ cells. Sidak’s test: B16F10+βGE*Sc* vs. B16F10 = 0.0032 (unstimulated), indicated by (**), and = 0.0125 (stimulated), indicated by (**). In **(C)**, the absolute number of CD3+CD8+IFN-γ+ cells. Sidak’s test: B16F10+βGE*Sc* vs. B16F10 = 0.0034, indicated by (**), (unstimulated) and = 0.0037 (stimulated), indicated by (**). In **(D)**, the absolute number of CD3+CD8+TNF-α+ cells. Sidak’s test: B16F10+βGE*Sc* vs. B16F10 = 0.0021 (unstimulated), indicated by (**), and = 0.0294 (stimulated), indicated by (**). Statistical differences between groups were assessed by using a one-way ANOVA followed by Sidak’s multiple-comparison test. Results are presented as mean ± SD. n = 4 per group. Statistical significance levels are indicated as follows: p < 0.05 (*), p < 0.01 (**), p < 0.001 (***), and p < 0.0001 (****).

The analysis of the absolute numbers of myeloid and lymphoid cells within the total splenocyte population is presented in **Figure 2**, following the definition of a specific cell selection strategy (**Supplementary Files S2, S3**). There is an increase in the absolute number of CD11b+ and total F4/80+ (single-positive) cells in animals treated with β-GESc in the presence (or not) of the tumor (**Figures 2A, B**). **Figure 2C** shows the number of F4/80+ cells within the CD11b+ total cell population, and the number of double-positive CD11b+F4/80+ splenic macrophages also increased in β-GE*Sc*-treated animals compared to the other experimental groups. In **Figures 2D**, mice treated with β-GESc showed increases in dendritic cells (CD11b+CD11c+) compared to the untreated groups. Additionally, in **Figures 2E**, higher expression of MHC class II was further observed in total CD11b+CD11c+ cells in β-GE*Sc*-treated mice. Additionally, the absolute numbers of natural killer (NK) cells (NK1.1+CD3−) and NKT (NK1.1+) cells were also examined. NK1.1+CD3+ cells increased in treated animals (**Figures 2F, G**), with a significant increase in the β-GE*Sc*-treated group in the absence of tumor. Untreated tumor-bearing groups have shown lower CD4+ and CD8+ T cell counts (**Figures 2H, I**) than the other experimental groups. According to our analysis strategy (see **Supplementary File S4**), β-GE*Sc*-treated animals showed higher percentages of CD4+IFNγ+ (**Figure 3A**) and TNF-α+CD4+ T cells (**Figure 3B**). CD8+ splenic T cells producing IFN-γ and TNF-α were also higher in treated animals, regardless of anti-CD3 stimulation (respectively **Figures 3C, D**). Higher IFN-γ and TNF-α production was also observed in both CD4+ and CD8+ T cells in β-GESc-treated mice, as indicated by relative numbers and shown in dot plots, compared to untreated controls (**Supplementary Files S5, S6**).

### 3.3 The treatment with β-GE*Sc* also led to an increase in mononuclear peripheral blood cells

**Figure 4:**
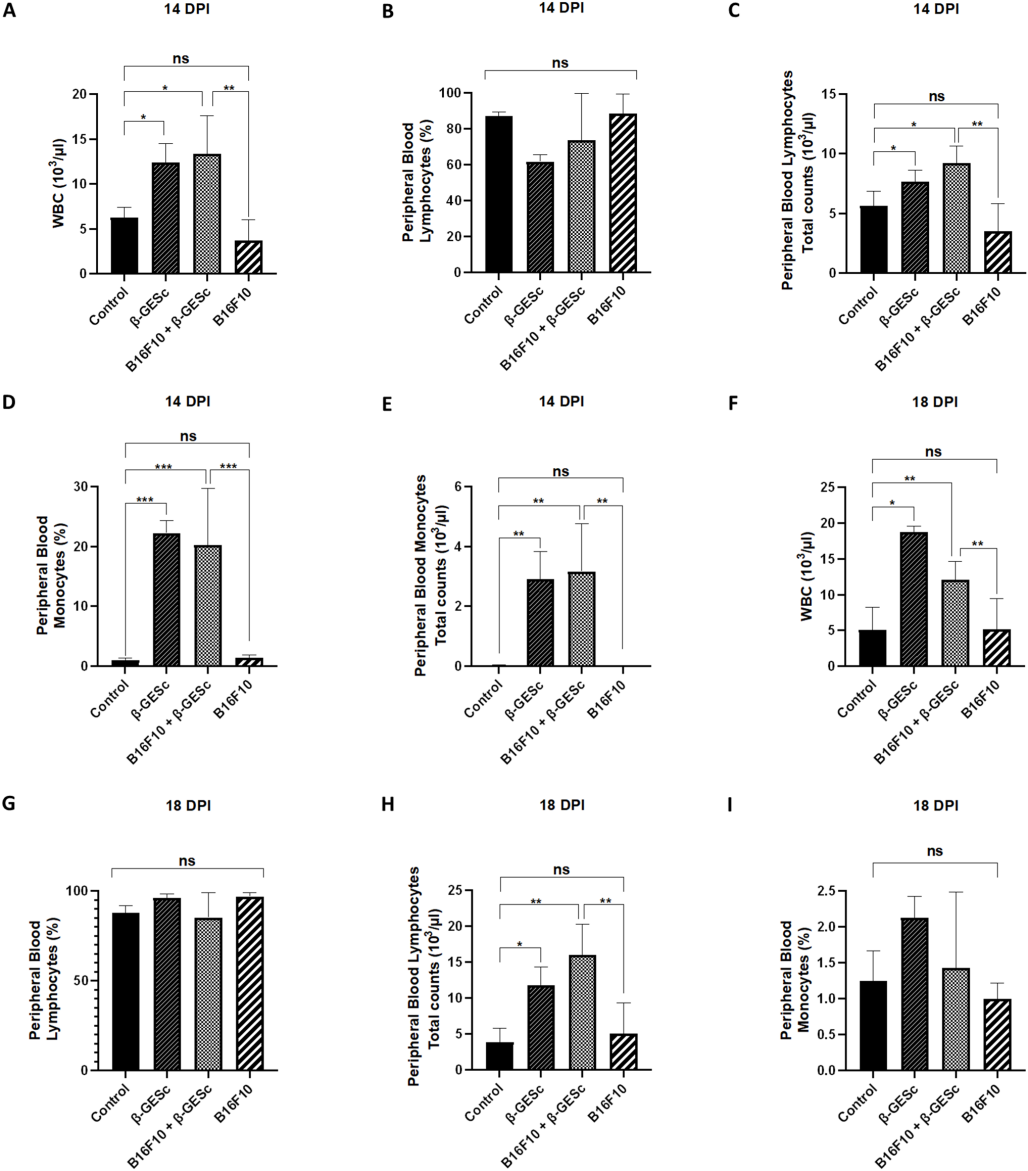
Peripheral blood cell analysis. In **(A)**, the absolute number of WBCs in peripheral blood at 14 days post-inoculation (d.p.i.), ANOVA = 0.0006 (p-value). Tukey's multiple comparisons: B16F10+GE*Sc* vs. B16F10 = 0.0014, indicated by (**). In **(B)**, the relative percentage of lymphocytes, ANOVA = 0.0658 (ns). In **(C)**, the absolute number of lymphocytes in peripheral blood, ANOVA = 0.0014; Tukey's multiple comparisons: B16F10+GE*Sc* vs. B16F10 = 0.0012, indicated by (**). In **(D)**, the percentage of monocytes, ANOVA < 0.0001. Tukey's multiple comparisons: B16F10+GE*Sc* vs. B16F10 = 0.0007, indicated by (***). In **(E)**, the absolute number of monocytes in peripheral blood, ANOVA < 0.0003. Tukey's multiple comparisons: B16F10+GE*Sc* vs. B16F10 = 0.0020, indicated by (**). In **(F)**, WBC count at 18 d.p.i., ANOVA < 0.0001. Tukey's multiple comparisons: B16F10+GE*Sc* vs. B16F10 = 0.0280, indicated by (*). In **(G)**, lymphocyte percentage, ANOVA = 0.3925. In **(H)**, the absolute number of lymphocytes, ANOVA = 0.0008; Tukey's comparison: B16F10+GE*Sc* vs. B16F10 = <0.0031, indicated by (**), In **(I)**, monocyte percentage at 18 d.p.i., ANOVA = 0.1007; Tukey's comparison: B16F10+GE*Sc* vs B16F10 = <0.7504 (ns). Statistical differences between groups were assessed by one-way ANOVA followed by Tukey's multiple comparison test. Results are presented as mean ± SD. n = 5 per group. Statistical significance levels are indicated as follows: p < 0.05 (*), p < 0.01 (**), p < 0.001 (***), and p < 0.0001 (****).

Systemic effects of β-GE*Sc* therapy or treatment were observed in **Figure 4**, revealing a significant increase in circulating leukocytes (WBC) in the peripheral blood of the treated groups (**Figure 4A****).** Additionally, the absolute number of total lymphocytes is increased in treated animals (**Figure 4C**). At 14 days post-inoculation (d.p.i.), significant changes were noted in both relative (**Figure 4D**) and absolute monocyte counts (**Figure 4E**) in the treated animals. By the assessment at 18 d.p.i., the effects of β-GE*Sc* treatment became even more pronounced in total lymphocytes (**Figure 4H**), particularly in the absolute numbers of circulating peripheral cell blood populations (**Figure 4F**). Interestingly, the relative values of monocytes at this assessment point and the absolute monocyte numbers also returned to baseline (data not shown).

### 3.4 Animals with tumors exhibit a high presence of melanin-expressing cells and disorganization of the structural compartments in the spleen

**Figure 5:**
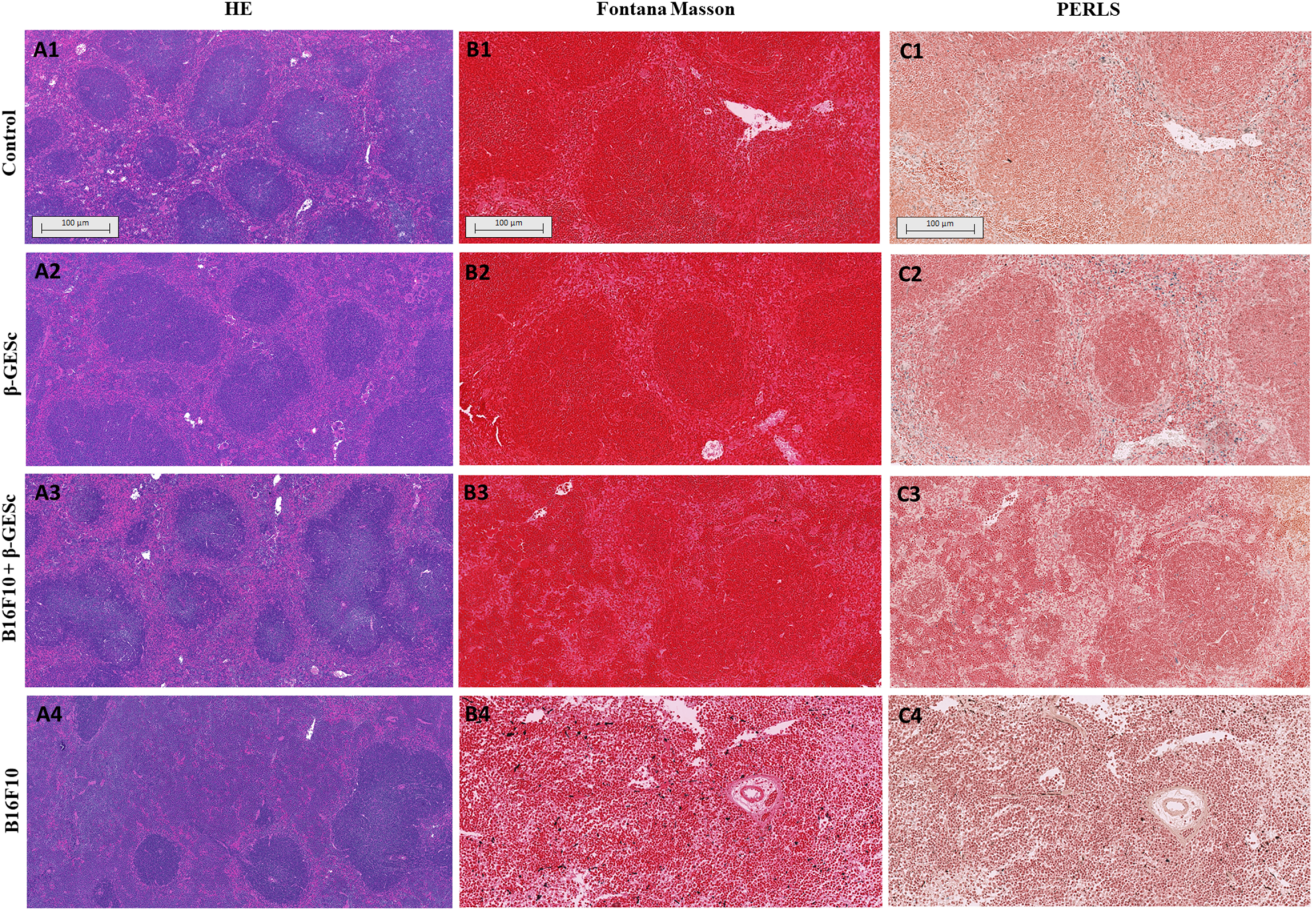
Representative photomicrographs of the spleen from C57BL/6 mice were submitted for histological evaluation 18 days post-inoculation (d.p.i.). In **(A1)**, representative micrographs of splenic tissue from intact control animals were stained with hematoxylin and eosin (H&E), 100nm. In **(A2)**, representative micrographs of only animals treated with βGE*Sc* were stained with H&E, 100nm. In **(A3)**, representative micrographs of tumor-bearing animals treated with βGE*Sc*, H&E staining, 100nm. In **(A4)**, representative micrographs of tumor-bearing animals without treatment, H&E staining, 100nm. In **(B1–B4)**, representative photomicrographs of melanin analysis by Fontana-Masson staining (FM) for each experimental group are shown. In **(C1–C4)**, representative photomicrographs of iron analysis by Perls’ Prussian blue staining.

Histological analysis on 18 d.p.i identified structural compartments in the splenic tissue, particularly in areas populated by B and T lymphocytes, as seen in **Figure 5**. Animals treated *in vivo* with β-GE*Sc* maintain a significantly preserved area of white pulp, both in the absence of the tumor (**Figure 5-A2**) and in tumor-bearing mice (**Figure 5-A3**). In contrast, untreated mice carrying tumors (the B16F0 group) exhibit significant disorganization of the splenic architecture, characterized by destruction and reduction of the white pulp, as shown in **Figure 5-A4**.

### 3.5 The reduction of white pulp in tumor-bearing animals is attributed to decreased areas of B and T cells, while β-GE*Sc* treatment helps maintain the structural splenic organization

**Figure 6:**
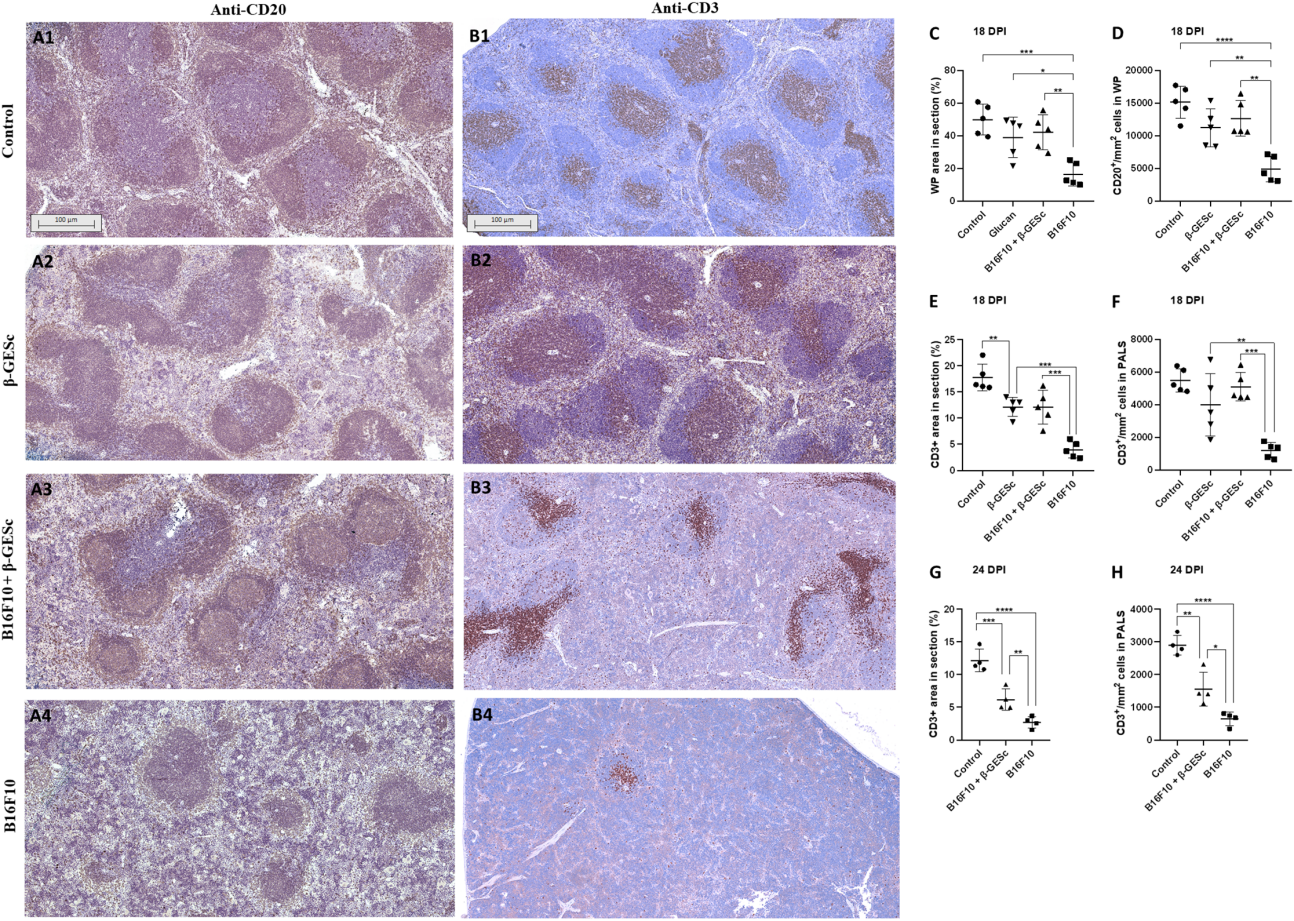
Representative photomicrographs of IHC with anti-CD20 and anti-CD3 for evaluating white pulp (WP) regions. In **(A1-A4)**, photomicrographs of areas (100nm) labeled with anti-CD20 represent the experimental groups at 18 d.p.i. In **(B1-B4)**, photomicrographs of regions labeled with anti-CD3 represent the evaluated groups at 18 d.p.i. In **(C)**, evaluation of the percentage of white pulp (WP) area marked in the total splenic section at 18 d.p.i., ANOVA = <0.0001. Tukey's multiple comparisons: B16F10+GESc vs. B16F10 = 0.0048, indicated by (**). **(D)** Analysis of the number of CD20+ cells within the lymphoid follicle at 18 d.p.i., ANOVA = 0.0009. Tukey's multiple comparisons: B16F10+GESc vs. B16F10 = 0.0010, indicated by (**). In **(E)**, evaluation of the percentage of CD3+ cell area marked in the total splenic section at 18 d.p.i., ANOVA = <0.0001. Tukey's multiple comparisons: B16F10+GESc vs. B16F10 = 0.0003, indicated by (***). **(F)** Analysis of the number of CD3+ cells within the PALS region at 18 d.p.i., ANOVA = <0.0001. Tukey's multiple comparisons: B16F10+GESc vs. B16F10 = 0.0003, indicated by (***). In **(G)**, evaluation of the percentage of CD3+ cell area marked in the total splenic section at 24 d.p.i., ANOVA = <0.0001. (H) Analysis of the number of CD3+ cells within the PALS region at 24 d.p.i., ANOVA = <0.0001.

Splenic mitotic activity in unstructured tissues was observed **in** **Figure 5**, along with marked melanin deposits, specifically in untreated tumor-bearing mice at 24 d.p.i (**Figure 5-B4**). We also confirmed that the presence of these pigments is not related to tissue iron content (**Figure 5-C4**). On the other hand, tumor-bearing animals that were β-GE*Sc*-treated did not display melanin deposits throughout the evaluated splenic sections (**Figure 5-B3**). A pronounced reduction in the white pulp in the untreated group-bearing tumors is evident in **Figure 5-A4.6** treatment during experimental melanoma development. **Figure 6** shows that an anti-CD20 or anti-CD3 monoclonal antibody (mAb) was used to identify B or T cells in splenic tissue. When compared to control mice in **Figure 6 A1-A2** (β-GE*Sc* treated or not), **Figure 6-A3** shows that tumor-bearing animals treated with β-GE*Sc* maintained the CD20+ cell area, which was distinguished by the presence of germinal centers (GCs), indicative of immune activation. Conversely, untreated tumor-bearing mice showed a reduced area marked by CD20+ cells when compared to the other experimental groups (**Figure 6-A4****).** The morphometric analysis in **Figure 6C** confirmed that animals bearing tumors without β-GE*Sc* treatment showed a reduction in the percentage of areas marked by CD20+ cells compared to the other groups. Similarly, the number of follicular CD20+ cells is lower, as shown in **Figure 6D**.

Regarding T cells, untreated tumor-bearing animals (compared to the other groups) showed significant disruption of the periarteriolar lymphoid sheath (PALS) region, with reduced T cells across all evaluated splenic sections, as illustrated in **Figure 6B4**. Compared to the control β-GE*Sc* treated and the B16F10 β-GE*Sc* treated mice, a decreased area marked by CD3+ was also observed at 18 d.p.i in **Figure 6E**. Additionally, this reduced area (**Figure 6E**) correlated with lower absolute counts in the T cell regions identified by morphometric analysis (**Figure 6F**). Kinetics later revealed a smaller area occupied by CD3+ cells (**Figure 6G**) and lower T-cell counts (**Figure 6H**) in untreated animals. In contrast, treated animals exhibited higher numbers of T cell areas and individual T cells.

## 4 Discussion

High-molecular-weight β-glucan molecules with β-1,3 linkages have demonstrated potential to promote anti-tumor responses (42). Such yeast cell wall components are likely essential in innate immunity and the tumor response, acting as pathogen-associated molecular patterns (PAMPs) (17). These polysaccharides are mainly recognized by C-lectin receptors (Dectin-1, -2,-3), DC-SIGN, and Toll-like receptors (TLRs) (43), (44). Notably, Dectin-1, which possesses a β-(1,3)-glucan layer and activates Syk through dimerization, plays a crucial role in this process, as reviewed in (45, 46). Recent mechanistic reviews (46, 47) reaffirm that Dectin-1 is the main β-glucan recognition ligand and detail how its activation triggers the production of pro-inflammatory cytokines and the maturation of dendritic cells, including in antitumor responses, presenting findings observed in our melanoma model (48).

It is estimated that these molecules, in the cell wall of *S. cerevisiae*, correspond to between 15-20% of the cell’s dry volume, being composed mainly of β-glucan polysaccharides, which play essential roles in cell morphology, preservation of osmotic integrity, and in the stages of cell growth and reproduction (49, 50). In the dry weight of the cell wall, β1,3-glucans account for 50-55%, followed by β1,6-glucan (5-10%), mannoproteins (35-40%), and chitin (1-2%) (51). Thus, activating pattern recognition receptors (PRRs) with glucans can trigger signaling pathways that enhance cellular activation and release inflammatory mediators as reviewed by (52).

High-molecular-weight β-glucan molecules with β-1,3 linkages enhance tumor response (42). These polysaccharides are primarily recognized by C-type lectin PRRs, Dectin-1, 2-3, DC-SIGN (Dendritic cell-specific intercellular adhesion molecule-3-grabbing non-integrin), and toll-like receptors (TLRs) (43, 44). The main PRR involved in glucan-mediated signaling appears to be Dectin-1, a receptor with a type C extracellular lectin domain that binds to β-(1,3)-glucan, initiating intracellular signaling and connecting to a signaling tail through a transmembrane domain (46). Interestingly, studies also demonstrate the expression of Dectin-1 in epithelial cells, such as melanocytes and keratinocytes (53, 54), and primary human epithelial cells of the ileum and colon, which respond to glucans with an inflammatory response, promoting the release of various mediators (55). Significant immunological effects, largely driven by interactions between β-glucans and Dectin-1 and TLRs, are evident across different cells of the innate immune system. (56).

While the anti-tumor mechanisms of β-glucans are not yet fully understood, their efficacy in enhancing immune responses has been observed in various tumors such as lung, gastric, cervical, and colorectal (57–60). Another study identified the potential of the supernatant obtained from *Saccharomyces cerevisiae* to induce apoptosis and inhibit cell growth in colonic cancer cells (61). Also, the authors noted that this effect is attributed to β-glucan extracted from *S. cerevisiae* in hepatoma cells but is innocuous to other non-neoplastic cells. In the present study, the administration of β-GE*Sc* modulated the immune response in a murine model of melanoma inoculated into the pinna of the animals (**Supplementary Figure 1B**), resulting in increased spleen size and a greater absolute number of splenocytes (**Table 1**), both in the presence and absence of a tumor. These findings were correlated with increased survival rates and reduced tumor size in treated animals, highlighting a direct impact on tumor development.

Previous studies have demonstrated that β-glucan can activate and modulate myeloid cells, including macrophages, monocytes, and dendritic cells, as well as stimulate NK cells, which are essential for the anti-tumor response (reviewed in 62). In treated animals compared with untreated mice, we identified significantly higher numbers of single-positive CD11b+ myeloid cells and double-positive CD11b+F4/70+ cells, which were macrophages, DCs, and splenic NK and NKT cells (**Figure 2**). High-molecular-weight glucans can activate macrophages by being degraded into smaller fragments that effectively challenge tumor cells (63). The 1 mg/day dose of β-GESc was used based on a robust bibliographic base and previously validated experimental protocols for yeast β-glucans in murine models. Additionally, we included in the study that research using a 1 mg dose of β-glucan/animal demonstrated significant immunological responses, including activation, increasing pro-inflammatory cytokines such as TNF-α, and increased aerobic glycolysis in monocytes, mediated by mTOR/HIF1α as a metabolic basis, as reviewed by the authors (35, 36, 38). Previous studies have shown that the dose-effect range is concentrated between 40 and 80 mg/kg in mice, a biologically active and safe dose, with no evidence of systemic toxicity (64). After treatment with β-glucan and anti-PD-L1 monoclonal antibody, tumor-infiltrating leukocytes (TILs) not only showed competent T-cell function (CD107a, perforin, IL-2, IFN-γ, and Ki67) and CTL population, but also demonstrated increased activity of tumor-recruited CD11b+ cells (IL-12, IL-6, IL-1β, and PD-1) (65). As reviewed by Cohen‐Kedar et al. (55), recent clinical studies have shown promising results with the combination of β-glucans and immune checkpoint inhibitors in patients with different solid tumors. Additionally, treatment with β-glucan increases MHCII+ DCs, while combined therapy with checkpoint inhibitors optimizes the CD8+/Treg ratio, enhancing antitumor immunity (66).

More splenic antigen-presenting cells (APCs) were found in β-GE*Sc*-treated mice. In fact, monocytes, macrophages, and DCs are crucial for inducing immune responses against tumors (67). These cells are increasingly utilized in cellular immunotherapy strategies, particularly for reprogramming tumor-associated macrophages (TAMs) (68). Here, we also observed an increase in splenic DCs expressing high levels of MHC class II. These cells can function as a vaccine or adjuvant in cancer inhibition (69). Notably, significant expression of Dectin-1 has been identified in the spleen and thymus, particularly in specialized DCs and Langerhans cells, which can synergistically interact with T lymphocytes (70). Consequently, these findings highlight the importance of investigating the role of microbial particles and their interactions in activating of immunological pathways that can combat tumors (46).

Natural killer (NK) cells can directly lyse tumor cells (71, 72). These cells infiltrate various tissues and induce a cytotoxic response (71). Our results demonstrated an increase in the absolute number of NK cells (NK1.1+/CD3-) in treated animals, along with a rise in the number of NKT cells (**Figure 2F**). This increase may correlate with the positive clinical results observed in melanoma-affected animals undergoing treatment, as NK cells in splenic tissue can contribute to T-cell polarization and the production of pro-inflammatory cytokines such as IFN-γ (73). Moreover, NKT cells can trigger significant anti-tumor responses via CD1d (74).

Although CD4+ and CD8+ T cells do not increase directly in splenic tissue, these cells can release IFN-γ, especially after stimulation with anti-CD3 (**Figure 3**). IFN-γ is also associated with increased MHC expression, which aids in presentation efficiency (75). Additionally, TNF-α synthesis was significantly higher in treated animals, both in the absence and presence of anti-CD3 stimulation, potentially contributing to an effective response against the tumor, especially after a secondary stimulus (**Figures 3B, D**). We argue that the optimal response is achieved with administration in the initial days after tumor implantation in our model. These TNF-α molecules are directly linked to robust anti-tumor responses (76), and they can induce apoptosis in tumor cells by interacting with specific receptors (77).

In addition to the increase in splenic cells, we observed a substantial rise in total blood leukocytes in treated animals at 14 days post-inoculation (d.p.i.) (**Figure 4**), accompanied by an increase in the absolute numbers of both lymphocytes and monocytes. The percentage of leukocytes and total lymphocytes (**Figures 4F–H**) increases at a more advanced stage of tumor development, while monocyte numbers return to normal. These results suggest that β-glucan has a stimulatory effect, promoting the release of cells from the bone marrow into the peripheral blood, thereby increasing their availability (78). Furthermore, the splenic structure appears indicative of extramedullary hematopoiesis or increased numbers of regulatory or myeloid suppressor cells in the present study.

Regarding structural aspects, disorganization of the splenic compartments is typically observed in cases of infectious disease (41), but there is limited data regarding the behavior of these structures in tumors. In the data obtained herein, we observed a considerable reduction in the white pulp (WP) areas throughout the spleen sections (**Figure 5A**) at 18 days post-injection (d.p.i.), particularly in tumor-bearing animals that were not treated. Given the spleen’s role in protecting against circulating pathogens, the disruption of spleen architecture may contribute to increased susceptibility to coinfections in patients with cancer. In contrast, β-GE*Sc*-treated animals maintained white pulp regions dispersed throughout the section (**Figure 5-A2**, **5-A3**). Furthermore, we noted melanin pigment accumulation in the spleens of untreated tumor-bearing animals, suggesting tumor cell infiltration into splenic tissue (**Figure 5-B4**). In β-GE*Sc*-treated animals, such pigmentation did not accumulate. Although splenic disorganization can occur due to melanoma, the spleen is not typically considered a common site for metastasis, which is rare and late (79). Histopathological evaluation by two independent pathologists, revealed no malignant alterations suggestive of metastasis in the liver or lungs. In some animals - predominantly in the untreated group - we observed mild intrasinusoidal leukocytosis in the liver, without additional histopathological alterations in the lungs across the analyzed groups. However, discrepant melanin deposits were observed in β-GE*Sc* untreated mice. Therefore, B16 melanoma appears to develop preferentially in the lungs following endovenous injection Hof tumor cells, where B16 tumor-cells are introduced directly into the bloodstream, facilitating rapid and predictable dissemination to the lungs or liver (80, 81).

Additionally, patients with intra-abdominal metastases may develop splenic metastasis (82). These results may be associated with the increased number of immune cells observed in both the spleen (**Figure 2**) and peripheral blood (**Figure 4**), which contribute to the tumor´s effector response. Specific staining with anti-CD20 and anti-CD3 antibodies confirmed that the reduction in white pulp is due to the atrophy of both the B and T cell areas (**Figure 6**). This reduction is particularly significant in untreated tumor-bearing animals, affecting both B cells and T cells. In contrast, β-GE*Sc*-treated tumor-bearing animals exhibit larger regions of B cells and T lymphocytes and show the presence of germinal center (GC) regions, indicating an enhanced local immune response.

Once activated, lymphocytes can quickly circulate between the “light” zone of the GC (which facilitates T cell helper signaling) and the “dark” zone, where they undergo rapid proliferation, immunoglobulin class switchings, and somatic hypermutation (83). Morphometric analysis of the data revealed a decrease in the white pulp area throughout the section and a reduced area occupied by CD20+ cells in the follicular region. The area occupied by the T cell region (PALS) throughout the splenic section also decreased, along with the number of T cells within the PALS at 18 d.p.i (**Figure 6****).** By 24 d.p.i, the area occupied by T cells and the number of T cells in the PALS region remained higher in treated tumor-bearing animals than in untreated ones.

These T cells, located in the T cell zone (TCZ) or PALS, are essential for the splenic immune response (84). B cells also play a crucial role in the spleen’s adaptive response; they can capture antigens directly from other antigen-presenting cells (APCs) and migrate to establish direct contact with T cells, leading to T cell activation (37).

## 5 Final considerations

The data presented in this study show that β-glucan extract (β-GE*Sc*) acts as a potent immune modulator, increasing all cell populations studied. This evidence suggests that β-glucan is associated with an increased cellular effector response through trained immunity (85). These findings were associated with increased survival rates and tumor reduction in this experimental melanoma model. This effect is likely mediated by the heightened presence of immune cells in the spleen and bloodstream, elevated levels of interferon-gamma (IFN-γ) and tumor necrosis factor-alpha (TNF-α) produced by T cells, and the formation of germinal centers that support the adaptive immune response. In addition, β-GE*Sc-*treatment preserved splenic organization and inhibited tumor-cell infiltration of B16F10 cells to the spleen.

Furthermore, β-GE*Sc* treatment maintained the spleen´s organization and inhibited cellular infiltration and tumor-derived material deposits in this organ induced by B16F10 cells. Finally, our results, characterized by increased macrophage frequency (CD11b+ F4/80+), and *in vivo* evaluations, suggest that β-glucan can induce the activation and production of IFN-γ and TNF-α, which are the expected effector outcomes of β-glucan-induced transcriptional reprogramming. The literature demonstrates that β-glucan stimulation, in a Dectin-1-dependent manner, triggers dynamic epigenetic changes, including increased marking in regulatory elements and histone trimethylation at inflammatory gene promoters (22, 52). These chromatin modifications, including epigenetic and metabolic reprogramming in these cells, are likely the mechanism that allows polarization towards the M1 phenotype and the persistence of the effector response observed in our model (52, 86). Future studies using anti-PDL-1 antibodies offer promising perspectives on the modifications mentioned above following adjuvant stimulation with β-GESc. These results emphasize the subcutaneous inoculation model used in this study as a reliable and reproducible method for future investigations into the metastatic behavior of B16F10 cells. Thus, our findings complement previous research and reinforce the therapeutic potential of *S. cerevisiae* β-glucan in treating melanoma, paving the way for its application in statistically a significant manner during immunotherapy.

## Data Availability

The original contributions presented in the study are included in the article/**Supplementary Material**. Further inquiries can be directed to the corresponding author.
