# Supplementary material for: The impact of β-glucan yeast extract treatment on melanoma development, tumor-cell deposit infiltration, and immune response
Source: Front Immunol. 2026 Apr 20;17:1752221. doi: 10.3389/fimmu.2026.1752221 (PMC13136267; doi:10.3389/fimmu.2026.1752221)
Supplement: Supplementary file 1 [file DataSheet1.docx]

Supplementary Material

#
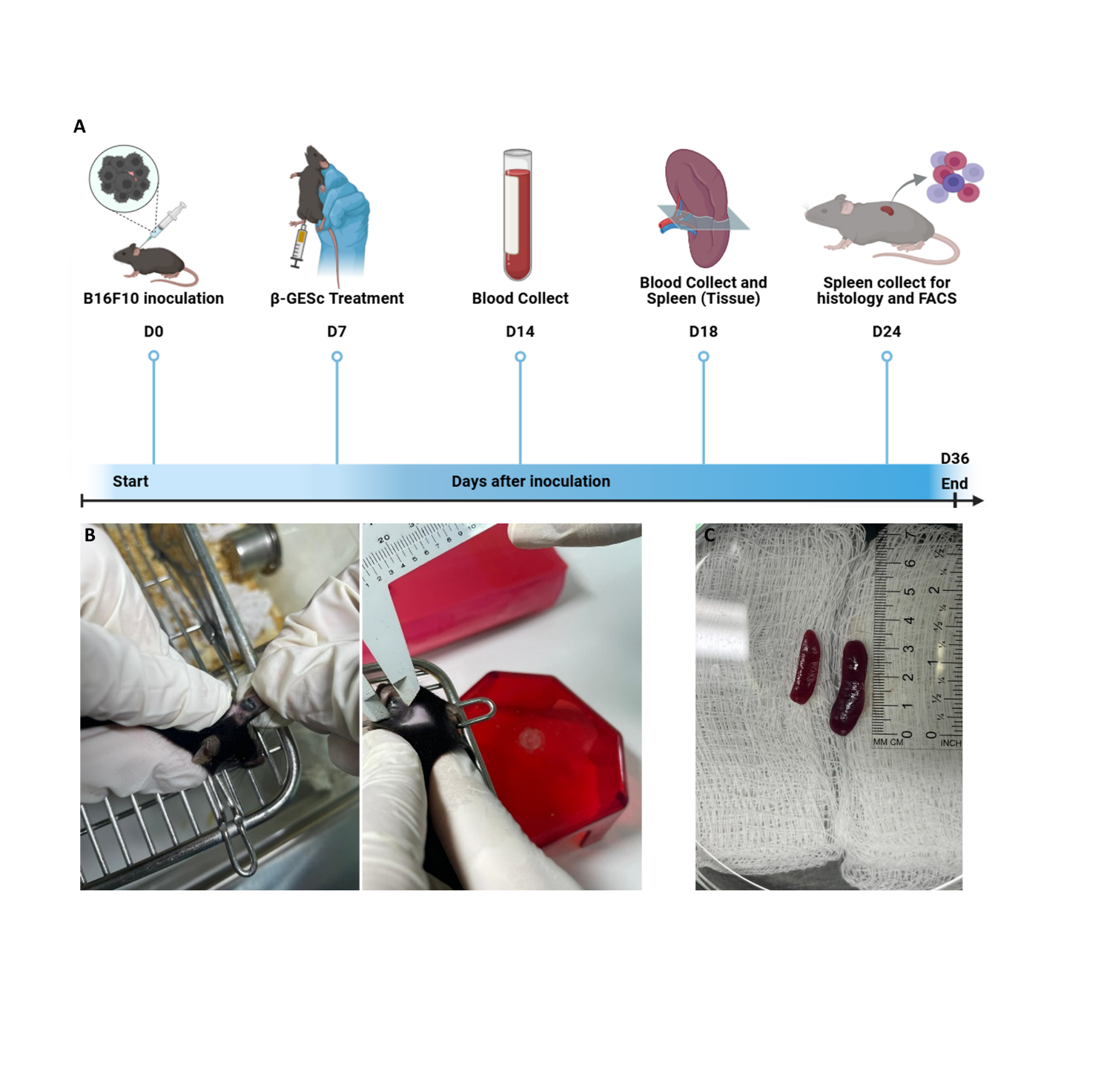
Supplementary Figures and Tables

**Supplementary Figure 1 (S1): Experimental protocol and representative images of the effect of treatment *in vivo*.** (A) Design and protocols of the timeline. (B) Representative images of the B16F10 cell injection site and the subcutaneous tumor growth pattern in the auricular pavilion of the animals. (C) Representative photograph of spleens from intact control animals (left) and animals treated only with β-GE*Sc* (right) at 24 days post-inoculation (d.p.i.).


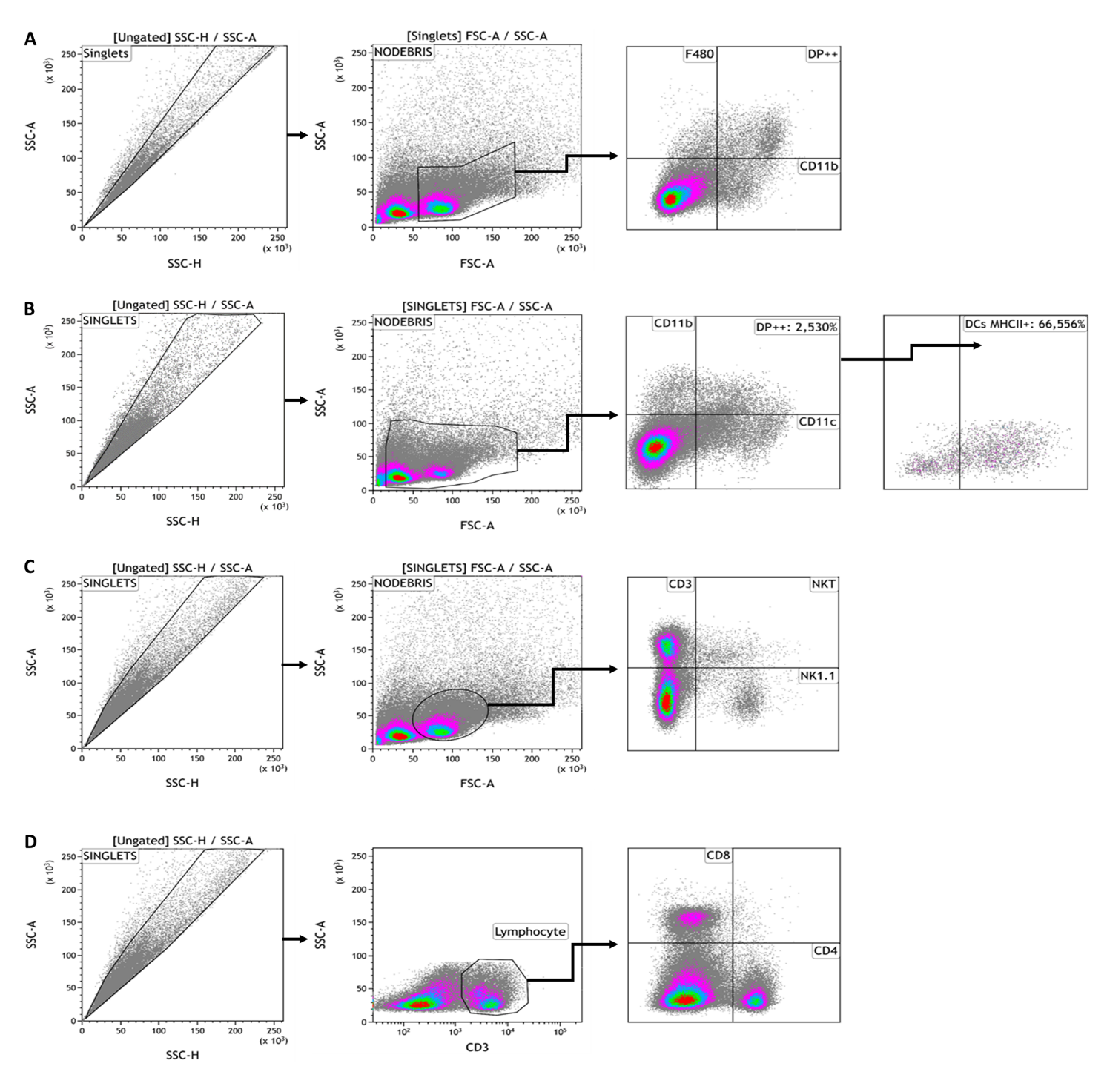
**Supplementary S2: Gating strategy used to analyze surface markers in selected cell populations.** S2A shows doublet cell-exclusion and cell selection gate for analysis of F4/80⁺CD11b⁺ cells. Doublet exclusion, cell selection gate for dendritic cell (DC) analysis, and representative dot plots of DC analysis and MHC class II expression in CD11b⁺CD11c⁺ cells are shown in S2B. In S2C, the gating strategy is used for analyzing NK and NKT cells. In S2D, a gating strategy is employed to evaluate CD3⁺/CD4⁺ and CD3⁺/CD8⁺ T lymphocytes. n=4/group. " Thanks, and sorry for any inconvenience this may cause, fabiola.


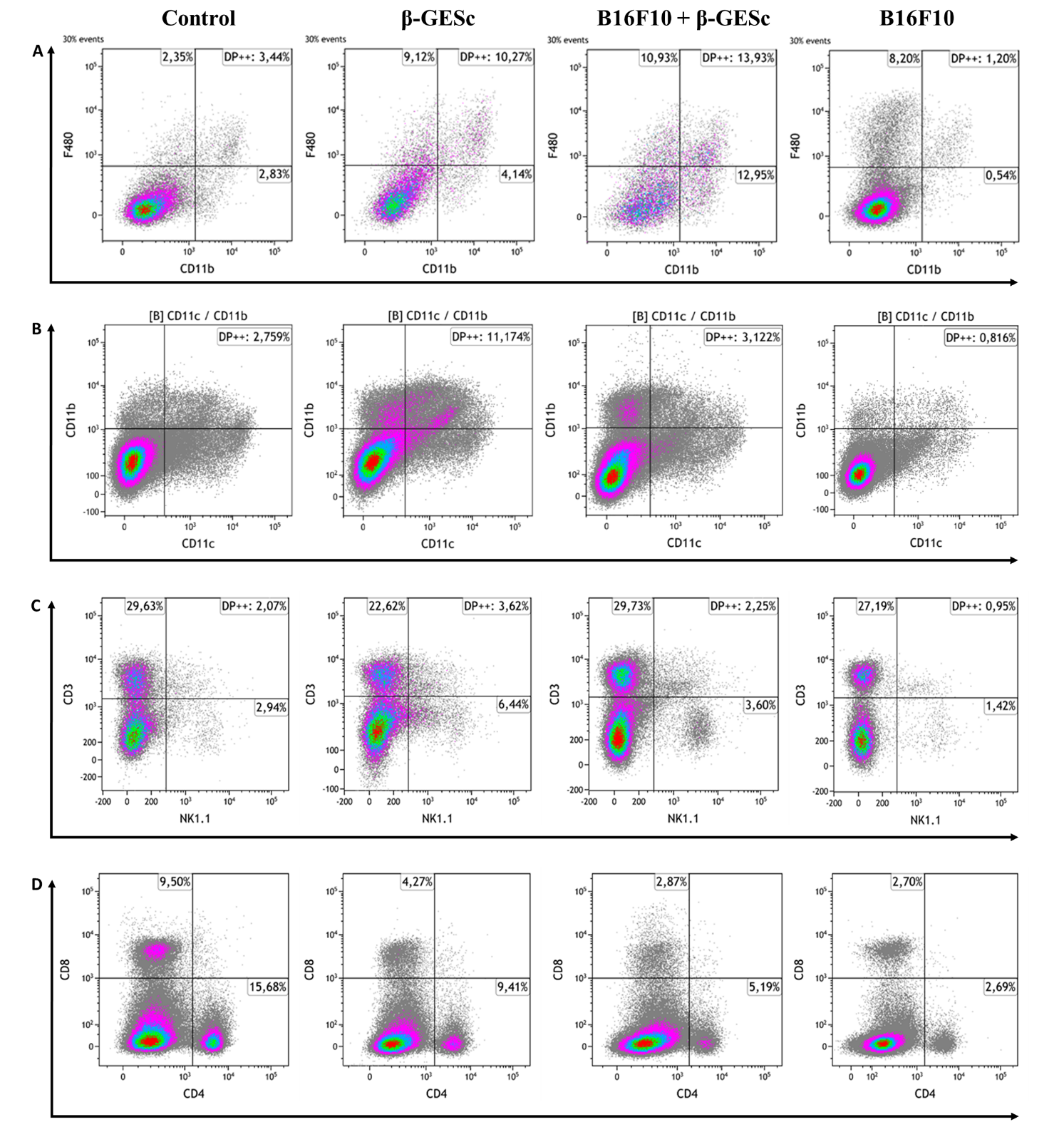
**Supplementary S3: Representative dot plots of the relative levels of surface markers in the experimental groups**. (A) Relative frequencies of F4/80⁺ (single-positive), CD11b⁺ (single-positive), and F4/80⁺/CD11b⁺ cells. (B) Relative frequencies of dendritic cells (CD11b⁺/CD11c⁺). (C) Relative frequencies of NK (CD3⁻/NK1.1⁺) and NKT (CD3⁺/NK1.1⁺) cells. (D) Relative frequencies of CD3⁺, CD4⁺ , and CD3⁺CD8⁺ T cells. n=4/group.
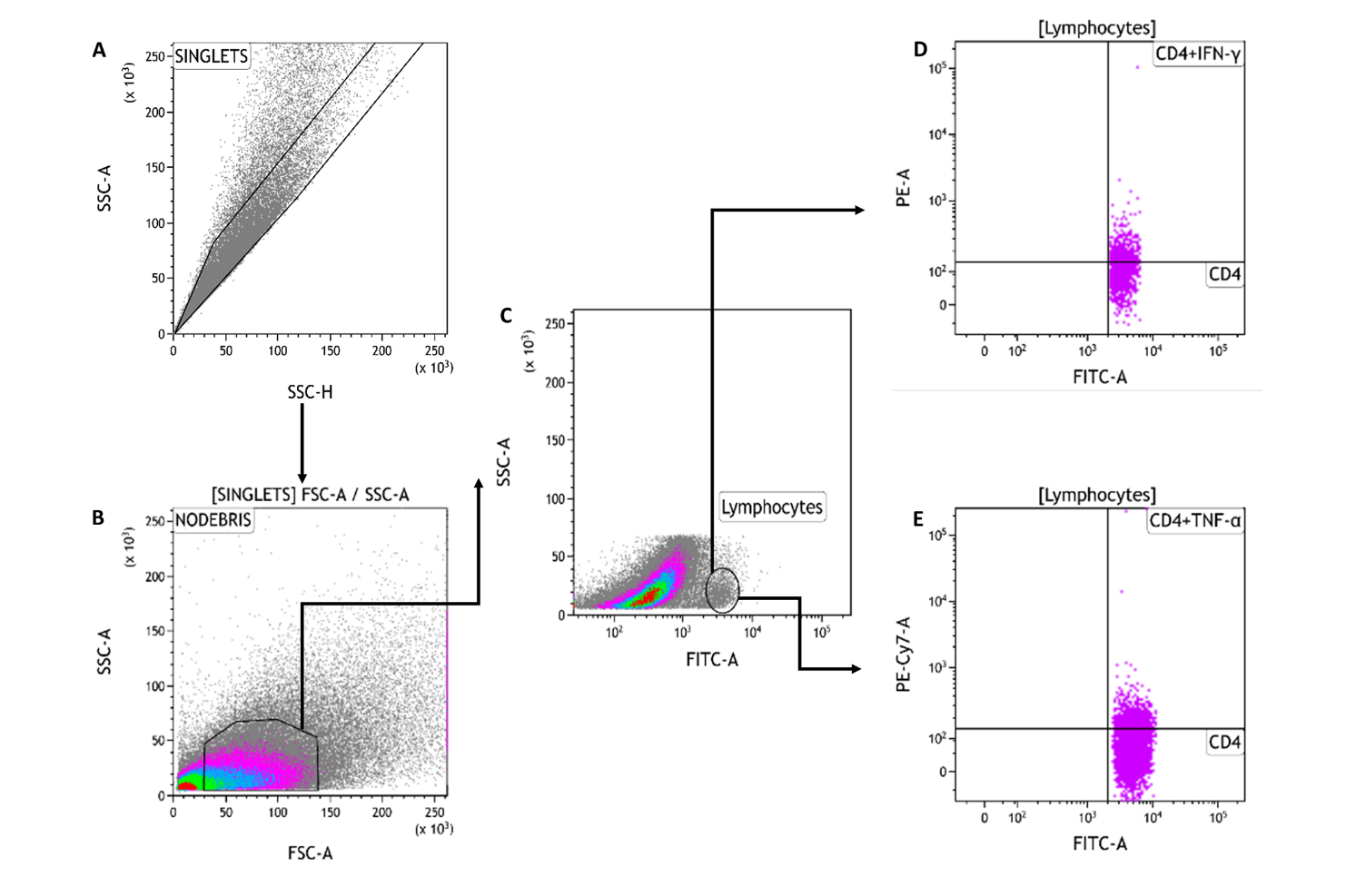
**Supplementary S4: Analysis strategy for IFN-γ and TNF-α production by CD3+ T cells.** Doublet cell-exclusion (A), cell selection gate (B), CD3+ cells gated (C), and representative cytokine dot plots of T-cell producers of IFN-γ (D) or TNF-α (E).


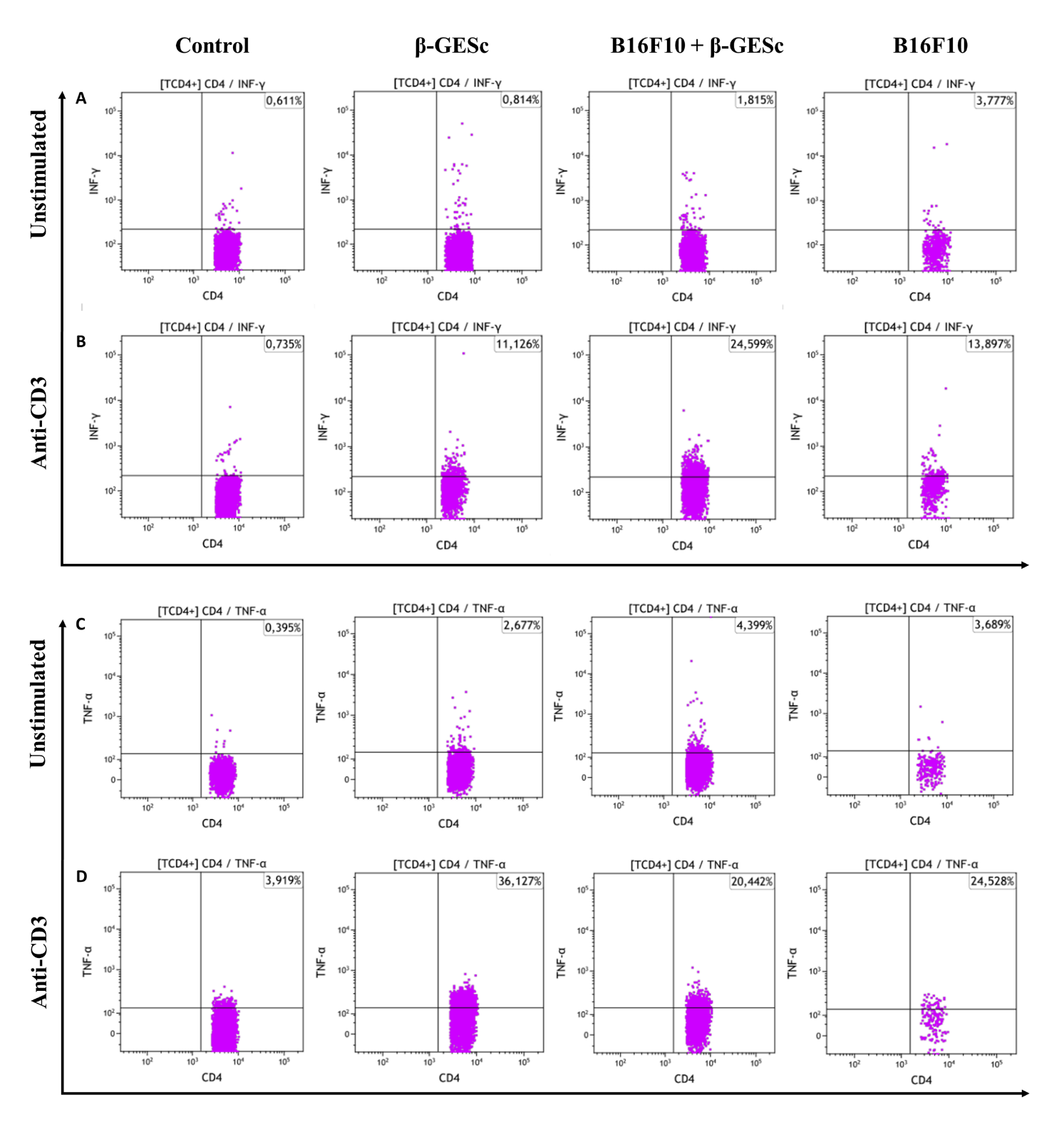
**Supplementary S5: Splenic CD3⁺/CD4⁺ and CD3⁺/CD8⁺ T cells.** (A-B) Representative dot plots of the experimental groups showing CD4⁺IFN-γ⁺ T cells in unstimulated cultures or in anti-CD3-stimulated cultures. (C-D), Representative dot plots of the experimental groups showing CD4⁺TNF-α⁺ T cells in unstimulated cultures or anti-CD3-stimulated cultures. n=4/group.


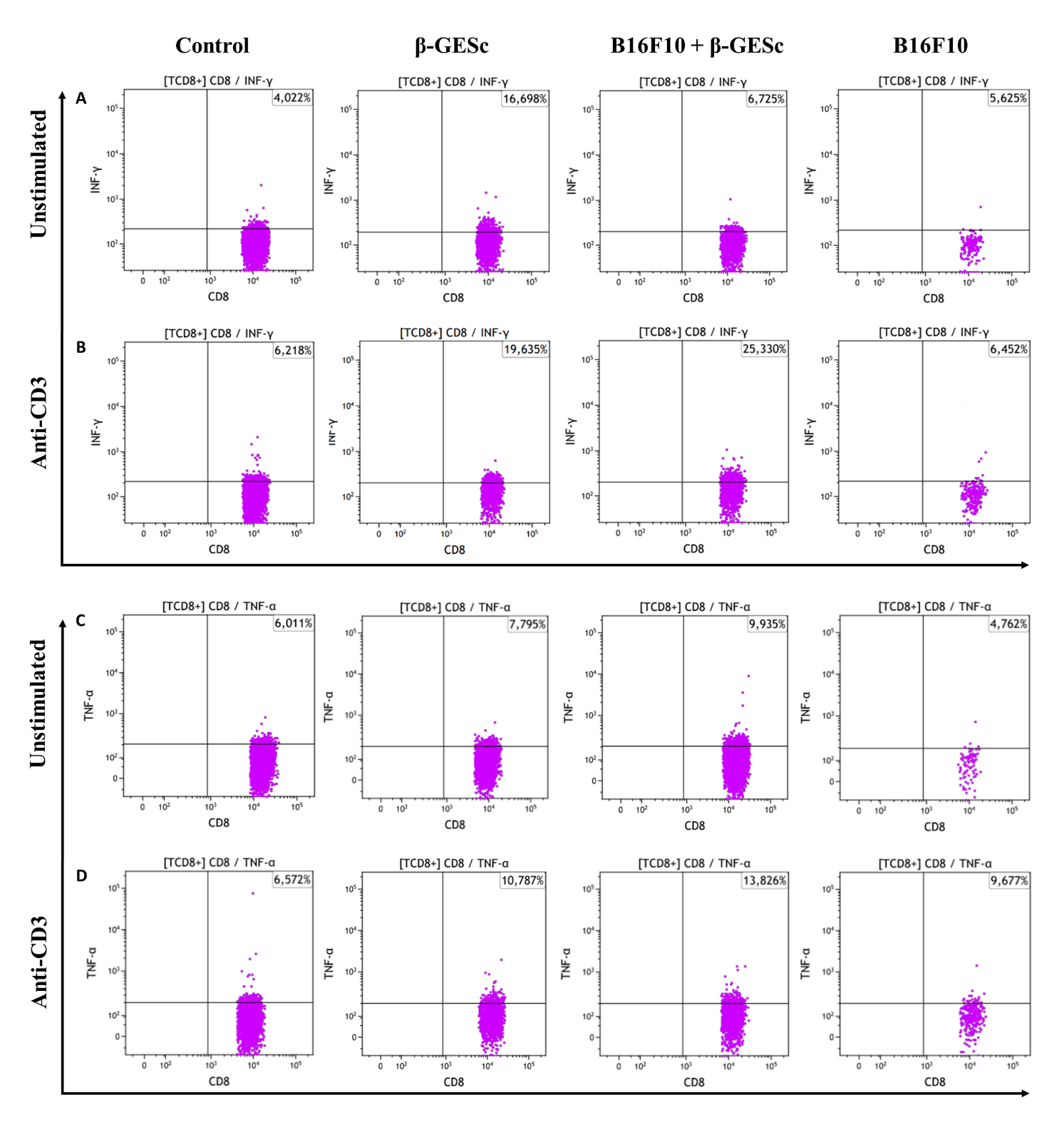


**Supplementary S6: Representative dot plots of TNF-α production by CD3⁺/CD4⁺ and CD3⁺/CD8⁺ T cells in experimental groups.** (A-B) Representative dot plots of the experimental groups showing CD8⁺IFN-γ⁺ T cells in unstimulated cultures or in anti-CD3-stimulated cultures. (C-D). Representative dot plots of the experimental groups showing CD8⁺TNF-α⁺ T cells in unstimulated cultures or anti-CD3-stimulated cultures. n=4/group.

**
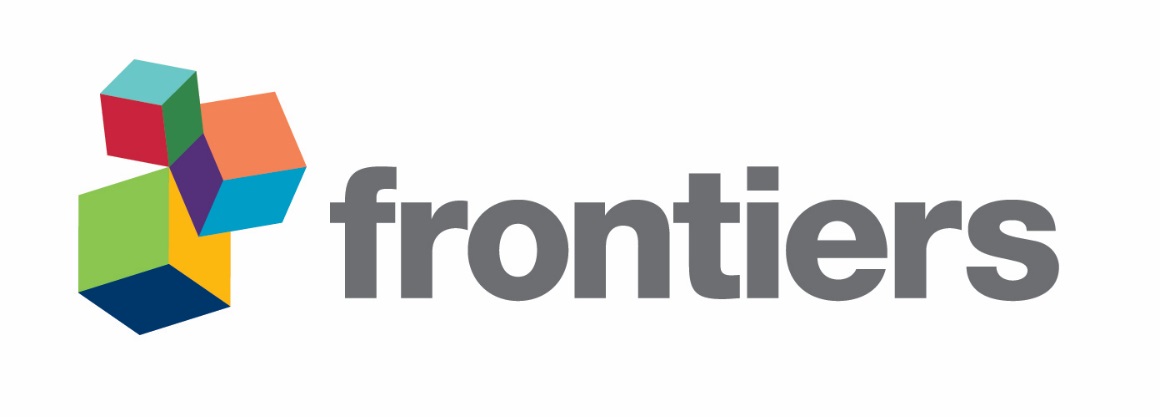
**
